# Physiology and Anatomy

**Published:** 1876-10

**Authors:** 


### V. Physiology and Anatomy.

1. *The Force of Ciliary Motion.* H. P. Bowditch, M.D. (*Boston M. and S. Jr.*, Aug. 10, 1876.)

After referring to the experiments of Valentine, Engelmann and Calimburces, demonstrating the power of the cilia to move very light bodies, B. describes the experiment of Prof. Wyman with heavier bodies. W. stretched, in a horizontal position, a patch of mucous membrane from the frog's mouth, previously dissected up, and having securely fixed it, he constructed a load, the under surface of which was made of fresh frog's skin, taken from near the throat. The rate at which this load was moved by the cilia was carefully noted, and with different sized weights. "A weight of 1.3 grammes was carried 15 m. m. in about one minute, the weight resting on a surface 12 m. m. square; and 48 grammes, resting on a surface 14 m. m. square," was moved but very slowly.

B. undertook to demonstrate the limit of the force capable of being exercised by cilia, and for the purpose arranged his experiments so as to compel the weight to be carried, instead of on a horizontal surface, up an inclined plane. "Then the product of the weight, by the height through which it was lifted, would give the value sought." This embodied the only essential modification of Wyman's experiments.

He took observations with the ciliated membrane inclined so as to make a grade of 10 per cent., afterward with the membrane vertical and with weights upon a base of 1.437 square c. m. of 5, 10 and 20 grammes "placed successively upon a vehicle weighing 0.534 gramme." The results, in brief, of his study show that the cilia on one square centimeter (4-10 inch square) of surface are capable of performing in one minute an amount of work equal to the lifting of 6.805 grammes (over 100 grs.) of weight through a height of one millimeter (1-25 inch). He concludes "the ciliated cells perform in one minute an amount of work equal to lifting their own weight to the height of 4.253 meters," (167⅓ inches.)

An idea of the rapidity of motion may be gained by noting that with the ciliated membrane vertical, and the weight about half a gramme (7 grs.), the rate at which the body was moved would have carried it through one inch of space in about two minutes and five seconds.

